# Supplementary material for: Impact of mental health stigma on help-seeking in the Caribbean: Systematic review
Source: PLoS One. 2023 Sep 12;18(9):e0291307. doi: 10.1371/journal.pone.0291307 (PMC10497129; doi:10.1371/journal.pone.0291307)
Supplement: S1 Appendix — (DOCX) [file pone.0291307.s002.docx]

##### S1 Appendix: Full database search strategies

These search strategies were developed for this review and follows this structure: Caribbean search terms AND stigma search terms AND mental health search terms.

**Note:** the key terms for the Caribbean are from a pre-tested search filter for the Caribbean Geographical Region Cited as:

Campbell, SM. Textword List to Retrieve Studies Related to the Caribbean Region from the OVID Medline Database. John W. Scott Health Sciences Library, University of Alberta. Rev. Feb 04, 2022. https://docs.google.com/document/d/1Wl_gusRPL9jLdDBJxhRQJPbPoQKEvLUOVC228T5z9xY/edi

**Search details**

| *Database* | *Through* | *Date of search* |
| --- | --- | --- |
| **EMBASE** | OvidSP | 13/05/2022 |
| **Medline** | OvidSP | 13/05/2022 |
| **Global Health** | Ovid SP | 13/05/2022 |
| **PsycInfo** | OvidSP | 13/05/2022 |
| **Scopus** |  | 13/05/2022 |
| **LILACS** |  | 18/05/2022 |

**EMBASE**

1. exp Caribbean/
2. exp Caribbean Community/
3. (Antigua or Antiguan* or Antilles or "Antillean Islands" or Aruba or Aruban* or Barbuda or Barbudan* or Bahamas or Bahamian* or Caribbean or Cuba or Cuban or Curacao or Curacaoan* or Dominica or Dominican* or "Dominican Republic" or Grenada or Grenadian* or Grenadines or Guadeloupe or Guadeloupean* or GuadeloupIan* or Haiti or Haitian or Jamaica* or Martinique or Martiniquais* or Martinican* or Nevis or Nevisian* or "Puerto Rico" or "Puerto Rican*" or "Saint Kitts" or Kittitian* or "Saint Lucia*" or "Saint Vincent" or Vincentian* or "Sint Maarten" or "Sint Maartener*" or Trinidad* or Trinidadian* or Tobago or Tobagonian* or "Virgin Island*" or "West Indies" or "West Indian*").mp. [mp=title, abstract, heading word, table of contents, key concepts, original title, tests & measures, mesh word]
4. 1 or 2 or 3
5. exp Attitudes/
6. exp Stereotyping/
7. exp Social Stigma/
8. exp Stigma/
9. exp Perception/
10. exp Prejudice/
11. stigma*.mp. [mp=title, abstract, heading word, table of contents, key concepts, original title, tests & measures, mesh word]
12. attitude*.mp. [mp=title, abstract, heading word, table of contents, key concepts, original title, tests & measures, mesh word]
13. discrimination.mp. [mp=title, abstract, heading word, table of contents, key concepts, original title, tests & measures, mesh word]
14. social distance.mp. [mp=title, abstract, heading word, table of contents, key concepts, original title, tests & measures, mesh word]
15. stereotyp*.mp. [mp=title, abstract, heading word, table of contents, key concepts, original title, tests & measures, mesh word]
16. prejudice*.mp. [mp=title, abstract, heading word, table of contents, key concepts, original title, tests & measures, mesh word]
17. exclusion.mp. [mp=title, abstract, heading word, table of contents, key concepts, original title, tests & measures, mesh word]
18. dangerousness.mp. [mp=title, abstract, heading word, table of contents, key concepts, original title, tests & measures, mesh word]
19. devaluation.mp. [mp=title, abstract, heading word, table of contents, key concepts, original title, tests & measures, mesh word]
20. label?ing.mp. [mp=title, abstract, heading word, table of contents, key concepts, original title, tests & measures, mesh word]
21. social acceptance.mp. [mp=title, abstract, heading word, table of contents, key concepts, original title, tests & measures, mesh word]
22. social perception.mp. [mp=title, abstract, heading word, table of contents, key concepts, original title, tests & measures, mesh word]
23. 5 or 6 or 7 or 8 or 9 or 10 or 11 or 12 or 13 or 14 or 15 or 16 or 17 or 18 or 19 or 20 or 21 or 22
24. exp Mental Health/
25. exp Mental Disorders/
26. mental health.mp. [mp=title, abstract, heading word, table of contents, key concepts, original title, tests & measures, mesh word]
27. mental disorder*.mp. [mp=title, abstract, heading word, table of contents, key concepts, original title, tests & measures, mesh word]
28. mental illness.mp. [mp=title, abstract, heading word, table of contents, key concepts, original title, tests & measures, mesh word]
29. depression.mp. [mp=title, abstract, heading word, table of contents, key concepts, original title, tests & measures, mesh word]
30. anxiety.mp. [mp=title, abstract, heading word, table of contents, key concepts, original title, tests & measures, mesh word]
31. bipolar.mp. [mp=title, abstract, heading word, table of contents, key concepts, original title, tests & measures, mesh word]
32. personality disorder*.mp. [mp=title, abstract, heading word, table of contents, key concepts, original title, tests & measures, mesh word]
33. schizophreni*.mp. [mp=title, abstract, heading word, table of contents, key concepts, original title, tests & measures, mesh word]
34. post?traumatic stress.mp. [mp=title, abstract, heading word, table of contents, key concepts, original title, tests & measures, mesh word]
35. ptsd.mp. [mp=title, abstract, heading word, table of contents, key concepts, original title, tests & measures, mesh word]
36. eating disorder*.mp. [mp=title, abstract, heading word, table of contents, key concepts, original title, tests & measures, mesh word]
37. "substance use".mp. [mp=title, abstract, heading word, table of contents, key concepts, original title, tests & measures, mesh word]
38. substance abuse.mp. [mp=title, abstract, heading word, table of contents, key concepts, original title, tests & measures, mesh word]
39. obsessive compulsive.mp. [mp=title, abstract, heading word, table of contents, key concepts, original title, tests & measures, mesh word]
40. ocd.mp. [mp=title, abstract, heading word, table of contents, key concepts, original title, tests & measures, mesh word]
41. 24 or 25 or 26 or 27 or 28 or 29 or 30 or 31 or 32 or 33 or 34 or 35 or 36 or 37 or 38 or 39 or 40
42. 4 and 23 and 41

**Medline**

1. exp Caribbean Region/

2. exp West Indies/

3. (Antigua or Antiguan* or Antilles or "Antillean Islands" or Aruba or Aruban* or Barbuda or Barbudan* or Bahamas or Bahamian* or Caribbean or Cuba or Cuban or Curacao or Curacaoan* or Dominica or Dominican* or "Dominican Republic" or Grenada or Grenadian* or Grenadines or Guadeloupe or Guadeloupean* or GuadeloupIan* or Haiti or Haitian or Jamaica* or Martinique or Martiniquais* or Martinican* or Nevis or Nevisian* or "Puerto Rico" or "Puerto Rican*" or "Saint Kitts" or Kittitian* or "Saint Lucia*" or "Saint Vincent" or Vincentian* or "Sint Maarten" or "Sint Maartener*" or Trinidad* or Trinidadian* or Tobago or Tobagonian* or "Virgin Island*" or "West Indies" or "West Indian*").mp. [mp=title, abstract, heading word, table of contents, key concepts, original title, tests & measures, mesh word]

4. 1 or 2 or 3

5. exp Attitude/

6. exp Stereotyping/

7. exp Social Stigma/

8. exp Prejudice/

9. exp Social Perception/

10. exp Social Discrimination/

11. stigma*.mp. [mp=title, abstract, heading word, table of contents, key concepts, original title, tests & measures, mesh word]

12. attitude*.mp. [mp=title, abstract, heading word, table of contents, key concepts, original title, tests & measures, mesh word]

13. discrimination.mp. [mp=title, abstract, heading word, table of contents, key concepts, original title, tests & measures, mesh word]

14. social distance.mp. [mp=title, abstract, heading word, table of contents, key concepts, original title, tests & measures, mesh word]

15. stereotyp*.mp. [mp=title, abstract, heading word, table of contents, key concepts, original title, tests & measures, mesh word]

16. prejudice*.mp. [mp=title, abstract, heading word, table of contents, key concepts, original title, tests & measures, mesh word]

17. exclusion.mp. [mp=title, abstract, heading word, table of contents, key concepts, original title, tests & measures, mesh word]

18. dangerousness.mp. [mp=title, abstract, heading word, table of contents, key concepts, original title, tests & measures, mesh word]

19. devaluation.mp. [mp=title, abstract, heading word, table of contents, key concepts, original title, tests & measures, mesh word]

20. label?ing.mp. [mp=title, abstract, heading word, table of contents, key concepts, original title, tests & measures, mesh word]

21. social acceptance.mp. [mp=title, abstract, heading word, table of contents, key concepts, original title, tests & measures, mesh word]

22. social perception.mp. [mp=title, abstract, heading word, table of contents, key concepts, original title, tests & measures, mesh word]

23. 5 or 6 or 7 or 8 or 9 or 10 or 11 or 12 or 13 or 14 or 15 or 16 or 17 or 18 or 19 or 20 or 21 or 22

24. exp Mental Health/

25. exp Mental Disorders/

26. mental health.mp. [mp=title, abstract, heading word, table of contents, key concepts, original title, tests & measures, mesh word]

27. mental disorder*.mp. [mp=title, abstract, heading word, table of contents, key concepts, original title, tests & measures, mesh word]

28. mental illness.mp. [mp=title, abstract, heading word, table of contents, key concepts, original title, tests & measures, mesh word]

29. depression.mp. [mp=title, abstract, heading word, table of contents, key concepts, original title, tests & measures, mesh word]

30. anxiety.mp. [mp=title, abstract, heading word, table of contents, key concepts, original title, tests & measures, mesh word]

31. bipolar.mp. [mp=title, abstract, heading word, table of contents, key concepts, original title, tests & measures, mesh word]

32. personality disorder*.mp. [mp=title, abstract, heading word, table of contents, key concepts, original title, tests & measures, mesh word]

33. schizophreni*.mp. [mp=title, abstract, heading word, table of contents, key concepts, original title, tests & measures, mesh word]

34. post?traumatic stress.mp. [mp=title, abstract, heading word, table of contents, key concepts, original title, tests & measures, mesh word]

35. ptsd.mp. [mp=title, abstract, heading word, table of contents, key concepts, original title, tests & measures, mesh word]

36. eating disorder*.mp. [mp=title, abstract, heading word, table of contents, key concepts, original title, tests & measures, mesh word]

37. "substance use".mp. [mp=title, abstract, heading word, table of contents, key concepts, original title, tests & measures, mesh word]

38. substance abuse.mp. [mp=title, abstract, heading word, table of contents, key concepts, original title, tests & measures, mesh word]

39. obsessive compulsive.mp. [mp=title, abstract, heading word, table of contents, key concepts, original title, tests & measures, mesh word]

40. ocd.mp. [mp=title, abstract, heading word, table of contents, key concepts, original title, tests & measures, mesh word]

41. 24 or 25 or 26 or 27 or 28 or 29 or 30 or 31 or 32 or 33 or 34 or 35 or 36 or 37 or 38 or 39 or 40

42. 4 and 23 and 41

**APA**

1. (Antigua or Antiguan* or Antilles or "Antillean Islands" or Aruba or Aruban* or Barbuda or Barbudan* or Bahamas or Bahamian* or Caribbean or Cuba or Cuban or Curacao or Curacaoan* or Dominica or Dominican* or "Dominican Republic" or Grenada or Grenadian* or Grenadines or Guadeloupe or Guadeloupean* or GuadeloupIan* or Haiti or Haitian or Jamaica* or Martinique or Martiniquais* or Martinican* or Nevis or Nevisian* or "Puerto Rico" or "Puerto Rican*" or "Saint Kitts" or Kittitian* or "Saint Lucia*" or "Saint Vincent" or Vincentian* or "Sint Maarten" or "Sint Maartener*" or Trinidad* or Trinidadian* or Tobago or Tobagonian* or "Virgin Island*" or "West Indies" or "West Indian*").mp. [mp=title, abstract, heading word, table of contents, key concepts, original title, tests & measures, mesh word]

2. exp Attitudes/

3. exp Stereotyped Attitudes/

4. exp Stigma/

5. exp Mental Health Stigma/

6. exp Discrimination/

7. exp Prejudice/

8. exp Social Perception/

9. stigma*.mp. [mp=title, abstract, heading word, table of contents, key concepts, original title, tests & measures, mesh word]

10. attitude*.mp. [mp=title, abstract, heading word, table of contents, key concepts, original title, tests & measures, mesh word]

11. discrimination.mp. [mp=title, abstract, heading word, table of contents, key concepts, original title, tests & measures, mesh word]

12. social distance.mp. [mp=title, abstract, heading word, table of contents, key concepts, original title, tests & measures, mesh word]

13. stereotyp*.mp. [mp=title, abstract, heading word, table of contents, key concepts, original title, tests & measures, mesh word]

14. prejudice*.mp. [mp=title, abstract, heading word, table of contents, key concepts, original title, tests & measures, mesh word]

15. exclusion.mp. [mp=title, abstract, heading word, table of contents, key concepts, original title, tests & measures, mesh word]

16. dangerousness.mp. [mp=title, abstract, heading word, table of contents, key concepts, original title, tests & measures, mesh word]

17. devaluation.mp. [mp=title, abstract, heading word, table of contents, key concepts, original title, tests & measures, mesh word]

18. label?ing.mp. [mp=title, abstract, heading word, table of contents, key concepts, original title, tests & measures, mesh word]

19. social acceptance.mp. [mp=title, abstract, heading word, table of contents, key concepts, original title, tests & measures, mesh word]

20. social perception.mp. [mp=title, abstract, heading word, table of contents, key concepts, original title, tests & measures, mesh word]

21. 2 or 3 or 4 or 5 or 9 or 10 or 11 or 12 or 13 or 14 or 15 or 16 or 17 or 18 or 19 or 20

22. exp Mental Health/

23. exp Mental Disorders/

24. mental health.mp. [mp=title, abstract, heading word, table of contents, key concepts, original title, tests & measures, mesh word]

25. mental disorder*.mp. [mp=title, abstract, heading word, table of contents, key concepts, original title, tests & measures, mesh word]

26. mental illness.mp. [mp=title, abstract, heading word, table of contents, key concepts, original title, tests & measures, mesh word]

27. depression.mp. [mp=title, abstract, heading word, table of contents, key concepts, original title, tests & measures, mesh word]

28. anxiety.mp. [mp=title, abstract, heading word, table of contents, key concepts, original title, tests & measures, mesh word]

29. bipolar.mp. [mp=title, abstract, heading word, table of contents, key concepts, original title, tests & measures, mesh word]

30. personality disorder*.mp. [mp=title, abstract, heading word, table of contents, key concepts, original title, tests & measures, mesh word]

31. schizophreni*.mp. [mp=title, abstract, heading word, table of contents, key concepts, original title, tests & measures, mesh word]

32. post?traumatic stress.mp. [mp=title, abstract, heading word, table of contents, key concepts, original title, tests & measures, mesh word]

33. ptsd.mp. [mp=title, abstract, heading word, table of contents, key concepts, original title, tests & measures, mesh word]

34. eating disorder*.mp. [mp=title, abstract, heading word, table of contents, key concepts, original title, tests & measures, mesh word]

35. "substance use".mp. [mp=title, abstract, heading word, table of contents, key concepts, original title, tests & measures, mesh word]

36. substance abuse.mp. [mp=title, abstract, heading word, table of contents, key concepts, original title, tests & measures, mesh word]

37. obsessive compulsive.mp. [mp=title, abstract, heading word, table of contents, key concepts, original title, tests & measures, mesh word]

38. ocd.mp. [mp=title, abstract, heading word, table of contents, key concepts, original title, tests & measures, mesh word]

39. 22 or 23 or 24 or 25 or 26 or 27 or 28 or 29 or 30 or 31 or 32 or 33 or 34 or 35 or 36 or 37 or 38

40. 1 and 21 and 39

**Global Health**

1. exp Caribbean/
2. exp Caribbean Community/
3. (Antigua or Antiguan* or Antilles or "Antillean Islands" or Aruba or Aruban* or Barbuda or Barbudan* or Bahamas or Bahamian* or Caribbean or Cuba or Cuban or Curacao or Curacaoan* or Dominica or Dominican* or "Dominican Republic" or Grenada or Grenadian* or Grenadines or Guadeloupe or Guadeloupean* or GuadeloupIan* or Haiti or Haitian or Jamaica* or Martinique or Martiniquais* or Martinican* or Nevis or Nevisian* or "Puerto Rico" or "Puerto Rican*" or "Saint Kitts" or Kittitian* or "Saint Lucia*" or "Saint Vincent" or Vincentian* or "Sint Maarten" or "Sint Maartener*" or Trinidad* or Trinidadian* or Tobago or Tobagonian* or "Virgin Island*" or "West Indies" or "West Indian*").mp. [mp=abstract, title, original title, broad terms, heading words, identifiers, cabicodes]
4. 1 or 2 or 3
5. exp Attitudes/
6. exp Social Stigma/
7. exp Discrimination/
8. exp Perception/
9. stigma*.mp. [mp=abstract, title, original title, broad terms, heading words, identifiers, cabicodes]
10. attitude*.mp. [mp=abstract, title, original title, broad terms, heading words, identifiers, cabicodes]
11. discrimination.mp. [mp=abstract, title, original title, broad terms, heading words, identifiers, cabicodes]
12. social distance.mp. [mp=abstract, title, original title, broad terms, heading words, identifiers, cabicodes]
13. stereotyp*.mp. [mp=abstract, title, original title, broad terms, heading words, identifiers, cabicodes]
14. prejudice*.mp. [mp=abstract, title, original title, broad terms, heading words, identifiers, cabicodes]
15. exclusion.mp. [mp=abstract, title, original title, broad terms, heading words, identifiers, cabicodes]
16. dangerousness.mp. [mp=abstract, title, original title, broad terms, heading words, identifiers, cabicodes]
17. devaluation.mp. [mp=abstract, title, original title, broad terms, heading words, identifiers, cabicodes]
18. label?ing.mp. [mp=abstract, title, original title, broad terms, heading words, identifiers, cabicodes]
19. social acceptance.mp. [mp=abstract, title, original title, broad terms, heading words, identifiers, cabicodes]
20. social perception.mp. [mp=abstract, title, original title, broad terms, heading words, identifiers, cabicodes]
21. 5 or 6 or 7 or 8 or 9 or 10 or 11 or 12 or 13 or 14 or 15 or 16 or 17 or 18 or 19 or 20
22. exp Mental Health/
23. exp Mental Disorders/
24. mental health.mp. [mp=abstract, title, original title, broad terms, heading words, identifiers, cabicodes]
25. mental disorder*.mp. [mp=abstract, title, original title, broad terms, heading words, identifiers, cabicodes]
26. mental illness.mp. [mp=abstract, title, original title, broad terms, heading words, identifiers, cabicodes]
27. depression.mp. [mp=abstract, title, original title, broad terms, heading words, identifiers, cabicodes]
28. anxiety.mp. [mp=abstract, title, original title, broad terms, heading words, identifiers, cabicodes]
29. bipolar.mp. [mp=abstract, title, original title, broad terms, heading words, identifiers, cabicodes]
30. personality disorder*.mp. [mp=abstract, title, original title, broad terms, heading words, identifiers, cabicodes]
31. schizophreni*.mp. [mp=abstract, title, original title, broad terms, heading words, identifiers, cabicodes]
32. post?traumatic stress.mp. [mp=abstract, title, original title, broad terms, heading words, identifiers, cabicodes]
33. ptsd.mp. [mp=abstract, title, original title, broad terms, heading words, identifiers, cabicodes]
34. eating disorder*.mp. [mp=abstract, title, original title, broad terms, heading words, identifiers, cabicodes]
35. "substance use".mp. [mp=abstract, title, original title, broad terms, heading words, identifiers, cabicodes]
36. substance abuse.mp. [mp=abstract, title, original title, broad terms, heading words, identifiers, cabicodes]
37. obsessive compulsive.mp. [mp=abstract, title, original title, broad terms, heading words, identifiers, cabicodes]
38. ocd.mp. [mp=abstract, title, original title, broad terms, heading words, identifiers, cabicodes]
39. 22 or 23 or 24 or 25 or 26 or 27 or 28 or 29 or 30 or 31 or 32 or 33 or 34 or 35 or 36 or 37 or 38
40. 4 and 21 and 39

**Scopus**

TITLE-ABS-KEY ( antigua OR antiguan* OR antilles OR "antillean islands" OR aruba OR aruban* OR barbuda OR barbudan* OR bahamas OR bahamian* OR caribbean OR cuba OR cuban OR curacao OR curacaoan* OR dominica OR dominican* OR "dominican republic" OR grenada OR grenadian* OR grenadines OR guadeloupe OR guadeloupean* OR guadeloupian* OR haiti OR haitian OR jamaica* OR martinique OR martiniquais* OR martinican* OR nevis OR nevisian* OR "puerto rico" OR "puerto rican*" OR "saint kitts" OR kittitian* OR "saint lucia*" OR "saint vincent" OR vincentian* OR "sint maarten" OR "sint maartener*" OR trinidad* OR trinidadian* OR tobago OR tobagonian* OR "virgin island*" OR "west indies" OR "west indian*" )

AND

TITLE-ABS-KEY ( stigma* OR attitude* OR discrimination OR "social distance" OR stereotyp* OR prejudice* OR exclusion OR dangerousness OR devaluation OR label?ing )

AND

TITLE-ABS-KEY ( "mental health" OR "mental disorder*" OR "mental illness" OR depression OR anxiety OR bipolar OR "personality disorder*" OR schizophreni* OR "post?traumatic stress" OR ptsd OR "eating disorder*" OR "substance use" OR "substance abuse" OR "obsessive compulsive" OR ocd)

**LILACS**

(TW:Antigua or Antiguan* or Antilles or "Antillean Islands" or Aruba or Aruban* or Barbuda or Barbudan* or Bahamas or Bahamian* or Caribbean or Cuba or Cuban or Curacao or Curacaoan* or Dominica or Dominican* or "Dominican Republic" or Grenada or Grenadian* or Grenadines or Guadeloupe or Guadeloupean* or Guadeloupean* or Haiti or Haitian or Jamaica* or Martinique or Martiniquais* or Martinican* or Nevis or Nevisian* or Nevisian*' or "Puerto Rican*" or "Saint Kitts" or Kittitian* or "Saint Lucia" or "Saint Vincent" or Vincentian* or "Sint Maarten" or "Sint Maartener" or Trinidad or Trinidadian or Tobago or Tobagonian or "Virgin Islands" or "West Indies" or "West Indian")

AND

(TW:stigma* OR attitude* OR discrimination OR “social distance” OR stereotyp* OR prejudice* OR exclusion OR dangerousness OR devaluation OR labeling OR “social acceptance” OR “social perception”)

AND

(TW:“mental health” OR “mental disorders” OR “mental illness” OR depression OR anxiety OR bipolar OR “personality disorder” OR schizophrenic OR “post traumatic stress” OR ptsd OR “eating disorders” OR "substance use" OR “substance abuse” OR “obsessive compulsive” OR OCD)
