# Supplementary material for: Impact of mental health stigma on help-seeking in the Caribbean: Systematic review
Source: PLoS One. 2023 Sep 12;18(9):e0291307. doi: 10.1371/journal.pone.0291307 (PMC10497129; doi:10.1371/journal.pone.0291307)
Supplement: S2 Appendix — (DOCX) [file pone.0291307.s003.docx]

**S2 Appendix: References for excluded papers (n=72) with primary reasons**

**Not data-based primary study (n=15)**

Burke AW. Trends in Social Psychiatry in the Caribbean. International Journal of Social Psychiatry. 1979;25(2):110–117. doi: 10.1177/002076407902500206

‌Compton B. Thinking Globally – Mental Illness: The burden of an unrecognized epidemic. Health Progress (Saint Louis, Mo.). 2013;94(2):68. Available from: https://www.chausa.org/docs/default-source/health-progress/997458f4139a42bc91cba85fc2fa7e4e1-pdf.pdf?sfvrsn=5ddf3af2_0

‌Francis KA, Molodynski A, Emmanuel G. Mental healthcare in Saint Lucia. BJPsych International. 2018;15(1):14–16. doi: 10.1192/bji.2017.12

‌Galli N. The influence of cultural heritage on the health status of Puerto Ricans. The Journal of School Health. 1975;45(1):10–16. doi: 10.1111/j.1746-1561.1975.tb04458.x

Ghali SB. Culture Sensitivity and the Puerto Rican Client. Social Casework. 1977;58(8):459–68. doi: 10.1177/104438947705800802

Hall N. Facing up to mental health problems. Mental Health Today (Brighton, England). 2015. Available from: <https://www.mentalhealthtoday.co.uk/facing-up-to-mental-health-problems>

Hickling FW, Gibson RC, Hutchinson G. Current research on transcultural psychiatry in the Anglophone Caribbean: Epistemological, public policy, and epidemiological challenges. Transcultural Psychiatry. 2013;50(6):858–75. doi: 10.1177/1363461513508806

Kobau R, Zack MM. Attitudes Toward Mental Illness in Adults by Mental Illness–Related Factors and Chronic Disease Status: 2007 and 2009 Behavioral Risk Factor Surveillance System. American Journal of Public Health. 2013;103(11):2078–89. doi: 10.2105/AJPH.2013.301321

Legha RK, Solages M. Child and Adolescent Mental Health in Haiti: Developing Long-Term Mental Health Services After the 2010 Earthquake. Child and Adolescent Psychiatric Clinics of North America. 2015;24(4):731–49. doi: 10.1016/j.chc.2015.06.004

Mascayano Tapia F, Lips W, Mena C, Manchego C. Stigma towards mental disorders: Characteristics and interventions. Salud Mental. 2015;38(1):53-58. doi: 10.17711/sm.0185-3325.2015.007

Piko BF. A new challenge: Depression is a significant problem among university students. In West Indian Medical Journal. 2009;58(1):1-2. Available from: <https://www.mona.uwi.edu/fms/wimj/system/files/article_pdfs/piko_a_new_challenge_depression_is_a_significant_problem.pdf>

Ramos-Olazagasti MA, Shrout PE, Yoshikawa H, Canino GJ, Bird HR. Contextual risk and promotive processes in Puerto Rican youths’ internalizing trajectories in Puerto Rico and New York. Development and Psychopathology. 2013;25(3):755–71. doi: 10.1017/S0954579413000151

Rayar O, Davies J. Cross‐cultural aspects of eating disorders in Asian girls. Nutrition & Food Science. 1996;96(4):19–22. doi: 10.1108/00346659610119207

Rosado JW. Important psychocultural factors in the delivery of mental health services to lower-class puerto rican clients: A review of recent studies. Journal of Community Psychology. 1980;8(3):215–26. doi: 10.1002/1520-6629(198007)8:3<215::AID-JCOP2290080304>3.0.CO;2-8

Stuber J, Meyer I, Link B. Stigma, prejudice, discrimination and health. Social Science & Medicine. 2008;67(3):351–7. doi: 10.1016/j.socscimed.2008.03.023

**Did not address mental health (n=3)**

Johnson D, Maguire ER, Maass SA, Hibdon J. Systematic observation of disorder and other neighborhood conditions in a distressed Caribbean community. Journal of Community Psychology. 2016;44(6):729–46. doi: 10.1002/jcop.21798

Oshi D, Abel W, Ricketts-Roomes T, Agu C, Oshi S, Harrison J, et al. Family structure, parental monitoring and marijuana use among adolescents in Jamaica: Findings from nationally representative data. West Indian Medical Journal. 2017;66(5):536-545. doi: 10.7727/wimj.2017.212

Payne MA. Barbadian children’s understanding of mental retardation. Applied Research In Mental Retardation. 1985; 6:185-193. doi: 10.1016/S0270-3092(85)80070-9

**Did not address stigma (n=12)**

Alegría M, Robles R, Freeman DH, Vera M, Jiménez AL, Ríos C, et al. Patterns of mental health utilization among island Puerto Rican poor. American Journal of Public Health. 1991;81(7):875–879. doi: 10.2105/AJPH.81.7.875

Arsuaga EN, Higgins JC, Sifre PA. Separation of brain-damaged from psychiatric patients with the combined use of an ability and a personality test: a validation study with a Puerto Rican population. Journal of Clinical Psychology. 1986;42(2):328–331. doi: 10.1002/1097-4679(198603)42:2<328::AID-JCLP2270420219>3.0.CO;2-X

Ayala RV. Link between eating disorder risk, self-esteem, and body image among puerto rican high school student-athletes. Journal of Physical Education and Sport. 2020;20(1):170-178. doi: 10.7752/jpes.2020.01023

Beckford Jarrett S, De La Haye W, Miller Z, Figueroa JP, Duncan J, Harvey K. High prevalence of psychiatric and substance use disorders among persons seeking treatment for HIV and other STIs in Jamaica: a short report. AIDS Care. 2017;30(5):604–8. doi: 10.1080/09540121.2017.1384786

Blanc J, Seixas A, Louis EF, Conserve DF, Casimir G, Jean-Louis G. Lessons Learned From a Low-Income Country to Address Mental Health Needs During COVID-19. Frontiers in Psychiatry. 2021;12(576352):1-4. doi: 10.3389/fpsyt.2021.576352

Brody EB. Psychocultural aspects of contraceptive behavior in jamaica individual fertility control in a developing country. Journal of Nervous and Mental Disease. 1974;159(2):108-119. doi: 10.1097/00005053-197408000-00005

Chen A, Smart Y, Morris-Patterson A, Katz CL. Piloting Self-Help Groups for Alcohol Use Disorders in Saint Vincent/Grenadines. Annals of Global Health. 2014;80(2):83-88. doi: 10.1016/j.aogh.2014.04.003

DelRosso LM, Cielo CM, D’Ulisse E, Elliot J, Galea L, Slavich L, et al. Efficacy of sleep education in a Dominican Republic neighborhood through training of community health promoters. Sleep Health. 2016;2(2):175–178. doi: 10.1016/j.sleh.2016.01.004

DeVylder JE, Kelleher I, Lalane M, Oh H, Link BG, Koyanagi A. Association of Urbanicity With Psychosis in Low- and Middle-Income Countries. JAMA Psychiatry. 2018;75(7):678-686. doi: 10.1001/jamapsychiatry.2018.0577

Lowe GA, Lipps G, Gibson RC, Halliday S, Morris A, Clarke N, et al. Neighbourhood factors and depression among adolescents in four Caribbean countries. PLoS ONE. 2014;9(4):1-14. doi: 10.1371/journal.pone.0095538

Vélez YD, Lorenzo-Luaces L, Rosselló J. Ideación suicida: Síntomas depresivos, pensamientos disfuncionales, autoconcepto, y estrategias de manejo en adolescentes puertorriqueños/as = Suicidal ideation: Depressive symptoms, dysfunctional thoughts, self-concept, and management strategies in Puert. Revista Puertorriqueña de Psicología. 2012;23:1-17. Available from: <https://www.redalyc.org/pdf/2332/233228917001.pdf>

Vera M, Alegría M, Freeman DH, Robles R, Pescosolido B, Peña M. Help Seeking for Mental Health Care Among Poor Puerto Ricans. Medical Care. 1998;36(7):1047–56. doi: 10.1097/00005650-199807000-00011

**Did not address help-seeking (n=5)**

Basden R, Khenti A. Attitudes toward persons who abuse drugs in one urban community on the island of new Providence, Bahamas. Texto e Contexto Enfermagem. 2019;28(Special Issue):1-12. doi: 10.1590/1980-265X-TCE-CICAD-7-13

Beck-Sague CM, Devieux JG, Pinzon-Iregui MC, Abreu-Perez R, Lerebours-Nadal L, Gaston S, et al. Depression in Caregivers of Status-Naive Pediatric HIV Patients Participating in a Status Disclosure Study in Haiti and the Dominican Republic: Preliminary Report. Journal of Tropical Pediatrics. 2015;61(1):65–68. doi: 10.1093/tropej/fmu060

Budhwani H, Hearld KR, Milner AN, Charow R, McGlaughlin EM, Rodriguez-Lauzurique M, et al. Transgender Women’s Experiences with Stigma, Trauma, and Attempted Suicide in the Dominican Republic. Suicide and Life-Threatening Behavior. 2018;48(6):788–796. doi: 10.1111/sltb.12400

Harrison AN, James C, Williams M, Gardner A, Scarlett SCD, Chang SM. 18. Adolescent Disordered Eating Behaviours and Attitudes in a Low-Middle Income Country. Journal of Adolescent Health. 2015;56(2):S10. doi: 10.1016/j.jadohealth.2014.10.021

Palmieri RG, Suárez Y. The future outlook of Puerto Rican Vietnam-era hospitalized psychiatric patients. Journal of Clinical Psychology. 1972;28(3):394-399. doi: 10.1002/1097-4679(197207)28:3+<394::AID-JCLP2270280326>3.0.CO;2-Q

**Did not address one of the research questions (n=15)**

Abel W, Longman-Mills S, Martin J, Oshi D, Whitehorne-Smith P. Does Ganja Cause Mental Illness? Perspectives from a Population-based Assessment of Mental Health Literacy in Jamaica. West Indian Medical Journal. 2017;66(5):553-561. [doi:](https://doi.org/10.7727/wimj.2017.209) 10.7727/wimj.2017.209

Allen D. Resocialization in The Bahamas: One family at a time. West Indian Medical Journal. 2016;65(Suppl 5):52-53. Available from: <https://www.mona.uwi.edu/fms/wimj/system/files/article_pdfs/7th_annual_research_day.pdf>

Arthur CM, Hickling FW, Robertson-Hickling H, Haynes-Robinson T, Abel W, Whitley R. “Mad, Sick, Head Nuh Good”: Mental Illness Stigma in Jamaican Communities. Transcultural Psychiatry. 2010;47(2):252–75. doi: 10.1177/1363461510368912

Bethell K, Allen D. Suicide in The Bahamas: Curbing a national epidemic. West Indian Medical Journal. 2016;65(Suppl 5):56. Available from: <https://www.mona.uwi.edu/fms/wimj/system/files/article_pdfs/7th_annual_research_day.pdf>

Cardeña E, Schaffler Y. "He Who Has the Spirits Must Work a Lot": A Psycho-Anthropological Account of Spirit Possession in the Dominican Republic. Ethos. 2018;46(4):457–76. doi: 10.1111/etho.12216

Hagaman AK, Wagenaar BH, McLean KE, Kaiser BN, Winskell K, Kohrt BA. Suicide in rural Haiti: Clinical and community perceptions of prevalence, etiology, and prevention. Social Science & Medicine. 2013;83:61–69. doi: 10.1016/j.socscimed.2013.01.032

Hansen H. The “new masculinity”: Addiction treatment as a reconstruction of gender in Puerto Rican evangelist street ministries. Social Science & Medicine. 2012;74(11):1721–1728. 10.1016/j.socscimed.2011.06.048

Hickling FW, Robertson-Hickling H, Paisley, V. Deinstitutionalization and attitudes toward mental illness in Jamaica: A qualitative study. Revista Panamericana de Salud Publica/Pan American Journal of Public Health. 2011;29(3):169-176). Available from: <https://www.scielosp.org/pdf/rpsp/2011.v29n3/169-176>

Keys HM, Kaiser BN, Foster JW, Burgos Minaya RY, Kohrt BA. Perceived discrimination, humiliation, and mental health: a mixed-methods study among Haitian migrants in the Dominican Republic. Ethnicity & Health. 2014;20(3):219–40. doi: 10.1080/13557858.2014.907389

Knight J, Gomez H, Perez M, Donastorg Y, Kerrigan D, Barrington C. 'I wish I had more help to be able to control myself with drugs': Experiences with substance use among female sex workers living with HIV in the Dominican Republic. Journal of the International AIDS Society. 2018; 21(S6 Suppl 6):146. doi: 10.1002/jia2.25148

Kohn R, Sharma D, Camilleri CP, Levav I. Attitudes towards mental illness in the Commonwealth of Dominica. Revista Panamericana de Salud Publica/Pan American Journal of Public Health. 2000;7(3):148-154. doi: 10.1590/S1020-49892000000300002

Rael CT, Davis A. Depression and key associated factors in female sex workers and women living with HIV/AIDS in the Dominican Republic. International Journal of STD and AIDS. 2017;28(5):433–440. doi: 10.1177/0956462416651374

Reid SD, Downes E, Khenti A. Participants’ perception of a unique community of practice for substance abuse education in the Caribbean. Substance Abuse. 2016;37(3):427–34. doi: 10.1080/08897077.2015.1134753

Williams DJ. Are Jamaicans really that stigmatizing? A comparison of mental health help-seeking attitudes. West Indian Medical Journal. 2013;62(5):437-442. doi: 10.7727/wimj.2013.074

Youssef FF, Bachew R, Bodie D, Leach R, Morris K, Sherma G. Knowledge and attitudes towards mental illness among college students: Insights into the wider English-speaking Caribbean population. International Journal of Social Psychiatry. 2014;60(1):47–54. doi: 10.1177/0020764012461236

**Ineligible population (n=10)**

Diefenbach GJ, Robison JT, Tolin DF, Blank K. Late-life anxiety disorders among Puerto Rican primary care patients: impact on well-being, functioning, and service utilization. Journal of Anxiety Disorders. 2004;18(6):841–858. doi: 10.1016/j.janxdis.2003.10.005

Gaviria M, Wintrob RM. Supernatural Influence in Psychopathology: Puerto Rican Folk Beliefs about Mental Illness. Canadian Psychiatric Association Journal. 1976;21(6):361–369. doi: 10.1177/070674377602100602

Graham PJ, Meadows CE. Psychiatric disorder in the children of West Indian immigrants. Journal of Child Psychology and Psychiatry. 1967;8(2):105–116. doi: 10.1111/j.1469-7610.1967.tb02186.x

Keating F, Robertson D. Fear, black people and mental illness: A vicious circle? Health and Social Care in the Community. 2004;12(5):439–447. doi: 10.1111/j.1365-2524.2004.00506.x

Lubdhansky I, Egri G, Stokes, J. Puerto Rican spiritualists view mental illness: the faith healer as a paraprofessional. The American Journal of Psychiatry. 1970;127(3):312-321. doi: 10.1176/ajp.127.3.312

Marcos LR, Uruyo L, Kesselman M, Alpert M. The Language Barrier in Evaluating Spanish-American Patients. Archives of General Psychiatry. 1973;29(5):655-659. doi: 10.1001/archpsyc.1973.04200050064011

Normand WC, Iglesias J, Payn S. Brief group therapy to facilitate utilization of mental health services by Spanish-speaking patients. American Journal of Orthopsychiatry. 1974;44(1):37–42. doi: 10.1111/j.1939-0025.1974.tb00866.x

Stacciarini JMR. Focus Groups: Examining a Community-Based Group Intervention for Depressed Puerto Rican Women. Issues in Mental Health Nursing. 2008;29(7):679–700. doi: 10.1080/01612840802128998

Weisenberg M, Kreindler ML, Schachat R, Werboff J. Pain: Anxiety and Attitudes in Black, White and Puerto Rican Patients. Psychosomatic Medicine. 1975;37(2):123–35. doi: 10.1097/00006842-197503000-00003

Wilder JF, Plutchnik R, Conte HR. Compliance With Psychiatric Emergency Room Referrals. Archives of General Psychiatry. 1977;34(8):930-933. doi: 10.1001/archpsyc.1977.01770200068006

**About views among professional caregivers (n=4)**

Caplan S, Little TV, Garces-King J. Stigma about mental illness among multidisciplinary health care providers in the Dominican Republic. International Perspectives in Psychology: Research, Practice, Consultation. 2016;5(3):192–206. doi: 10.1037/ipp0000057

Gardner W, Kelleher KJ, Wasserman R, Childs G, Nutting P, Lillienfeld H, et al. Primary Care Treatment of Pediatric Psychosocial Problems: A Study From Pediatric Research in Office Settings and Ambulatory Sentinel Practice Network. Pediatrics. 2000;106(4):1-9. doi: 10.1542/peds.106.4.e44

Negroni LK, Medina CK, Rivera Díaz M, Paniccia M. Perceptions of mental health and utilization of mental health services in Puerto Rico. Social Work in Mental Health. 2020;18(2):149–169. doi: 10.1080/15332985.2019.1700582

Winer RA, Morris-Patterson A, Smart Y, Bijan I, Katz CL. Knowledge of and Attitudes Toward Mental Illness Among Primary Care Providers in Saint Vincent and the Grenadines. Psychiatric Quarterly. 2013;84(3):395–406. doi: 10.1007/s11126-013-9254-6

**Wrong publication type** **(n=3)**

el Hamouti S. La carne de rené, a thesis novel. Anales de Literatura Hispanoamericana. 2018;47:447-454. doi: 10.5209/ALHI.62748

Govia I, Palmer T, Paisley-Clare V, Reynolds J, Edge D. Faith and spirituality in the Jamaican mental healthcare landscape. West Indian Medical Journal. 2017;66(Suppl 1):57-58. Available from: <https://www.mona.uwi.edu/fms/wimj/system/files/article_pdfs/carpha_2017_poster_abstracts_presentations_38-58.qxd_.pdf>

McShane KM. Mental Health in Haiti: A Resident’s Perspective. Academic Psychiatry. 2011;35(1):8–10. doi: 10.1176/appi.ap.35.1.8

**Could not retrieve (n=5)**

Devonish D. Managers’ perceptions of mental illness in Barbadian workplaces: an exploratory study. The Journal of Mental Health Training, Education and Practice. 2017;12(3):161–72. doi: 10.1108/JMHTEP-09-2016-0047

Gordon AJ. The cultural context of drinking and indigenous therapy for alcohol problems in three migrant Hispanic cultures. An ethnographic report. Journal of Studies on Alcohol. 1981;42(Suppl 9):217-249. doi: 10.15288/jsas.1981.s9.217

Hutchinson G, Neehall JE, Simeon DT, Littlewood R. Perceptions about mental illness among pre-clinical medical students in Trinidad & Tobago. The West Indian Medical Journal. 1999;48(2):81-84.

Mumford E. Puerto rican perspectives on mental illness. Mount Sinai Journal of Medicine. 1973;40(6):768-779.

Wray S. Prevalence and patterns of substance abusers: Neurobehavioural and social dimensions: a 1994 national survey report on substance abuse in Jamaica. 1994.
