# Supplementary material for: Impact of mental health stigma on help-seeking in the Caribbean: Systematic review
Source: PLoS One. 2023 Sep 12;18(9):e0291307. doi: 10.1371/journal.pone.0291307 (PMC10497129; doi:10.1371/journal.pone.0291307)
Supplement: S3 Appendix — (DOCX) [file pone.0291307.s004.docx]

**S3 Appendix: Full details and references for included papers**

This supplement outlines full sample and study characteristics for the quantitative (Table A) and qualitative (Table B) articles included in this review, followed by a list of full references for these.

**Table A.** Full sample and study characteristics for all quantitative articles included in the systematic review (n=6). Quality assessment (MMAT) = Number of criteria met on the Mixed Methods Assessment Tool.

| **Reference** | **Country** | **Study Design** | **n** | **Age** | **Sample Characteristics** | **Mental Health Condition of Study** | **Aspect of stigma** | **Aspect of help-seeking** | **Key Findings** | **Quality Assessment (MMAT)** |
| --- | --- | --- | --- | --- | --- | --- | --- | --- | --- | --- |
| Jackson Williams [1] | Jamaica | Cross-cultural survey | 339 | Mean age 17.18 years, Range 15-19 years | 146 Male and 193 Female Adolescents | Depression, Anxiety, Schizophrenia, Attention Deficit/Hyperactivity Disorder-Combined Type, Conduct Disorder, Eating Disorder Not Otherwise Specified and Substance Abuse | Public Stigma. Perceived Stigma | Attitudes | “…for a ‘psychological problem’…results indicate that students would seek help first from a medical doctor, followed by a faith healer and then from their teacher. Friends and family members were the last source of help…Across all disorders, with the exception of schizophrenia, students consistently identified friends and family members as their first choice of help. For schizophrenia, students reported that they would seek help from a psychologist/ psychiatrist first” (p467, 469) | 2/5 |
| Jackson Williams [2] | Jamaica | Cross-cultural survey | 339 | Range 15-19 years | 146 Male and 193 Female Adolescents | Not specified | Public Stigma | Attitudes | "results indicate that more negative opinions about mental illness, or more authoritarian and socially restrictive opinions, as well as less benevolent opinions were associated with less positive psychological help-seeking attitudes" (p371) | 3/5 |
| Maloney et al. [3] | Jamaica | Cross-cultural survey | 109 | Range 10-19 years | Survey 1 - 107 adolescents Survey 2 - 56 of the 107 adolescents from Survey 1 | Survey Part 1 - Not specified. Survey part 2 - Depression, Anxiety, Schizophrenia, Attention Deficit/Hyperactivity Disorder-combined type (ADHD), Conduct Disorder, Eating Disorder not otherwise specified, and Substance Abuse. | Public Stigma | Attitudes | "When asked to indicate barriers to seeking mental health care, respondents most frequently reported the problem was too personal/embarrassing...or not serious enough" (p4) | 2/5 |
| Nohr et al. [4] | Cuba | Cross-cultural survey | 195 (+ n=165 German participants) | Mean age 47.44 years | 59 Cuban Men and 136 Cuban Women | Not specified | Public Stigma | Attitudes | "community attitudes were a significant predictor of help-seeking attitudes" (p8) | 3/5 |
| Ramkissoon et al. [5] | Trinidad | Cross-cultural survey | 158 | Mean age 30 years | 136 Female and 22 Male University students | Schizophrenia | Public Stigma | Attitudes | "Significant associations existed between perceiving mental illness to be caused by supernatural factors, seeking religious/spiritual intervention... and seeking religious/spiritual intervention as the first in the health-seeking pathway" (p332) | 3/5 |
| Wageneer et al. [6] | Haiti | Cross-cultural survey | 408 | Not specified | 408 adults | Not specified | Public Stigma, Cultural Stigma | Intended | "Persons who stated that suffering from mental distress is never an individual’s fault were 3.5 times as likely as others to respond that they would turn to God first over hospitals or clinics and were .4 times as likely to respond that they would go to other community-based providers first compared with hospitals or clinics. Individuals responding that disasters can cause mental distress were 2.8 times as likely to respond that they would turn to God over hospitals or clinics." (pp368-369) | 2/5 |

**Table B.** Full sample and study characteristics for all qualitative articles included in the systematic review (n=3). Quality assessment (MMAT) = Number of criteria met on the Mixed Methods Assessment Tool.

| **Reference** | **Country** | **Study Design** | **n** | **Age** | **Sample Characteristics** | **Mental Health Condition of Study** | **Aspect of stigma** | **Aspect of help-seeking** | **Mental Health Condition of Study** | **Method of Analysis** | **Key Findings** | **Quality Assessment (MMAT)** |
| --- | --- | --- | --- | --- | --- | --- | --- | --- | --- | --- | --- | --- |
| Hannold et al. [7] | Puerto Rico | Semi-structured interview | 16 | Mean age 27 years for veterans, Mean age 36 years for family members | 8 veterans and 8 family members | Not Specified | Public Stigma | Attitudes | Not Specified | Qualitative Data Analysis | "Veterans may deny the need for psychological treatment because of stigma surrounding mental illness…FMs (family members) also perceived the stigma of mental illness to be real and problematic." (p385) | 4/5 |
| James et al. [8] | Jamaica | Semi-structured interview | 3 | Aged between 19-45 years | 3 case studies | Schizophrenia, Paranoid Schizophrenia, and Bipolar Disorder with Psychotic Features | Public Stigma, Cultural Stigma | Behaviours | Schizophrenia, Paranoid Schizophrenia, and Bipolar Disorder with Psychotic Features | Phenomenological Hermeneutic Approach | "The effects of the supernatural as the main cause of illness was a pervasive theme throughout the interviews. This in turn influenced the treatment that the individuals requested." (p259) | 5/5 |
| Liu et al. [9] | Saint Vincent and the Grenadines | Semi-structured interview | 30 | Not specified | 30 church leaders | Substance abuse - Alcoholism | Public Stigma | Attitudes | Substance abuse - Alcoholism | Grounded Theory Based Approach | "Those who had drinking problems tended to stray from and avoid the church, largely out of fear of condemnation: 'There are some sins members wouldn’t want to confess because it’s too shameful. Alcoholism is one of those issues.' " (p1086) | 3/5 |

**References for studies included in the review (n=9)**

[1] Jackson Williams D. Where do Jamaican Adolescents Turn for Psychological Help? Child Youth Care Forum. 2012;41(5):461-477. doi: 10.1007/s10566-012-9177-7

[2] Jackson Williams D. Help-Seeking Among Jamaican Adolescents: An Examination of Individual Determinants of Psychological Help-Seeking Attitudes. Journal of Black Psychology. 2014;40(4):359-383. doi: 10.1177/0095798413488940

[3] Maloney CA, Abel WD, McLeod HJ. Jamaican adolescents’ receptiveness to digital mental health services: A cross-sectional survey from rural and urban communities. Internet Interventions. 2020;21:1-9. doi: 10.1016/j.invent.2020.100325

[4] Nohr L, Ruiz AL, Sandoval Ferrer JE, Buhlmann U. Mental health stigma and professional help-seeking attitudes a comparison between Cuba and Germany. PLoS One. 2021;16(2):1-24. doi: 10.1371/journal.pone.0246501.

[5] Ramkissoon AK, Donald C, Hutchinson G. Supernatural versus medical: Responses to mental illness from undergraduate university students in Trinidad. International Journal of Social Psychiatry. 2017;63(4):330-338. doi: 10.1177/0020764017702412

[6] Wagenaar BH, Kohrt BA, Hagaman AK, McLean KE, Kaiser BN. Determinants of care seeking for mental health problems in rural haiti: Culture, cost, or competency. Psychiatric Services. 2013;64(4):366-372. doi: 10.1176/appi.ps.201200272

[7] Hannold EM, Freytes IM, Uphold CR. Unmet health services needs experienced by puerto rican OEF/OIF veterans and families post deployment. Military medicine. 2011;176(4): 381–388. doi: 10.7205/MILMED-D-10-00334

[8] James CCAB, Carpenter KA, Peltzer K, Weaver S. Valuing psychiatric patients’ stories: Belief in and use of the supernatural in the Jamaican psychiatric setting. Transcultural Psychiatry. 2014;51(2):247-263. doi: 10.1177/1363461513503879

[9] Liu S, Zafer M, Smart Y, Providence K, Katz CL. Knowledge of and Attitudes Toward Alcoholism Among Church Leaders in Saint Vincent/Grenadines. International Journal of Mental Health and Addiction. 2017;15(5):1081-1095. doi: 10.1007/s11469-017-9760-0
