# Supplementary material for: Impact of mental health stigma on help-seeking in the Caribbean: Systematic review
Source: PLoS One. 2023 Sep 12;18(9):e0291307. doi: 10.1371/journal.pone.0291307 (PMC10497129; doi:10.1371/journal.pone.0291307)
Supplement: S4 Appendix — (DOCX) [file pone.0291307.s005.docx]

**S4 Appendix: Themes and subthemes with example participant quotations for included qualitative studies**

The table below outlines the themes and subthemes that emerged from the thematic analysis undertaken to synthesise the findings of n=3 articles reporting qualitative data, alongside example participant quotations illustrating the subthemes.

| **Themes and subthemes** | **Exemplar quotations** |
| --- | --- |
| **Making sense of mental health conditions** |  |
| Labelling | "to me? I think that he is crazy, crazy…very crazy" [1] "I can't say that I am crazy - that I am suffering from that" [1] "I only get upset if somebody keep telling me I am mentally ill and know I am not mentally ill!...Once a psychotic, always a psychotic" [2] "I am not crazy" [2] |
| Sociocultural factors | "The culture nowadays among young people is that every week you go for a lime - liming meaning hanging out with friends - and drinking is a major part of it…you have to develop a counter-culture, one that makes drinking uncool, which is not easy to do, because the music, TV, everything points to alcohol" [3] "I prayed to God to please give me back my sanity, give me back my sanity, and he did and I got it back. I was insane for half an hour, insane. If it wasn't for God I would not have made it now because of this medication" [2] "Do you believe that witchcraft can start mental illness? It is in the Bible, you nuh, it in the Bible" [2] |
| Lack of recognition/denial of one’s own condition | "They [returning veterans] need mental help…that's because I know, I saw it. Mentally, there ae times…that there are times that you’re affected, and you don’t see it at first glance…" [1] "...sometimes there are people that are so closed up that they don't want...to say anything; I mean...I mean 'I am sane, I am alright.' What do I know?" [1] |
| **Anticipated/Experienced stigma-related experiences** |  |
| Social judgement | "So if you have something, you say to the people you have it in the church, you say to the people 'Come,' they will not come…Because they know that the church is a spiritual place, they think that when you come to them you will tell them, 'Stop it right now,' then they will not come. They think that you are going to preach and get them to stop using alcohol" [3] "There are some sins members wouldn't want to confess because it's too shameful. Alcoholism is one of those issues" [3] "Most of the users of alcohol are sometimes blatantly seen among the poor" [3] "'He came...came a Veteran and look - and came back crazy; came out crazy from the army.'...that they (neighbours/others) catalogue him like that..." [1] |
| Discrimination | "Or I want to get some job. I can't say that I am crazy - that I am suffering from that" [1] "A lot of alcoholics out there tend to turn into vagrants…so they lose family, they lose everything." [3] |
| Lack of understanding | "There are millions, millions of people around the world that are not paranoid schizophrenic that believe that they can bless their house and get rid of negative energy…And he thinks I'm hallucinating." [2] "The doctors say it was hallucination, but I know I wasn't hallucinating, for me that was an encounter with some supernatural stuff...to tell you the honest truth, I really don’t think I have an illness, because of how the spiritual aspect of life and the physical aspect of life [connect], I understand that because of this spiritual experience it leaves physical marks. That's where the doctors come in: they treat whatever marks, whatever scars exist" [2] |
| **Sociodemographic factors** |  |
| Military Personnel (characteristic) | "He came...came a Veteran and look - and came back crazy; came out crazy from the army.'...that they (neighbors/others) catalogue him like that..." [1] “I would say, we [Veterans] create our own support group. Our boys, we look for each other...talk to each other...meet with each other when we return from Iraq." [1] |

[1] Hannold EM, Freytes IM, Uphold CR. Unmet health services needs experienced by puerto rican OEF/OIF veterans and families post deployment. Military medicine. 2011;176(4): 381–388. doi: 10.7205/MILMED-D-10-00334

[2] James CCAB, Carpenter KA, Peltzer K, Weaver S. Valuing psychiatric patients’ stories: Belief in and use of the supernatural in the Jamaican psychiatric setting. Transcultural Psychiatry. 2014;51(2):247-263. doi: 10.1177/1363461513503879

[3] Liu S, Zafer M, Smart Y, Providence K, Katz CL. Knowledge of and Attitudes Toward Alcoholism Among Church Leaders in Saint Vincent/Grenadines. International Journal of Mental Health and Addiction. 2017;15(5):1081-1095. doi: 10.1007/s11469-017-9760-0
