# Supplementary material for: Impact of mental health stigma on help-seeking in the Caribbean: Systematic review
Source: PLoS One. 2023 Sep 12;18(9):e0291307. doi: 10.1371/journal.pone.0291307 (PMC10497129; doi:10.1371/journal.pone.0291307)
Supplement: Attachment — Submitted filename: Response to Reviewers.docx [file pone.0291307.s006.docx]

**PONE-D-23-14287**

Impact of mental health stigma on help-seeking in the Caribbean: systematic review

**Journal requirements:**

1. Please ensure that your manuscript meets PLOS ONE's style requirements, including those for file naming. The PLOS ONE style templates can be found at

https://journals.plos.org/plosone/s/file?id=wjVg/PLOSOne_formatting_sample_main_body.pdf and

https://journals.plos.org/plosone/s/file?id=ba62/PLOSOne_formatting_sample_title_authors_affiliations.pdf

**AUTHORS’ RESPONSE:** Thank you for this comment. We have reviewed the style templates and confirm our manuscript meets PLOS ONE’s style requirements.

2. Please review your reference list to ensure that it is complete and correct. If you have cited papers that have been retracted, please include the rationale for doing so in the manuscript text, or remove these references and replace them with relevant current references. Any changes to the reference list should be mentioned in the rebuttal letter that accompanies your revised manuscript. If you need to cite a retracted article, indicate the article’s retracted status in the References list and also include a citation and full reference for the retraction notice.

**AUTHORS’ RESPONSE:** Thank you for this comment. We have reviewed our reference list and confirm that it is complete and correct.

**AUTHORS’ COMMENT:** In reviewing the manuscript, we have noted that the line numbers were not continuous throughout the entire document. Additionally, the affiliation for Author PCG required correcting (page 1, line 6). Both have been amended and are reflected in the track changes.

Lastly, authors CS, GT, TTS and PCG all received financial support during the time they contributed to this work, which has been disclosed under the ‘Acknowledgements’ section in the Manuscript (page 24, lines 431-444) as shown in track changes.

**Additional Editor Comments:**

Abstract-Conclusion:

“This can be applied in the design of culturally appropriate future research, policy, and practice, to target and decrease stigma, and increase help-seeking in the Caribbean.”

This statement should be modified. As it is implies that the authors said this can be used to design future research and also design policy, and practice.

My suggestion:

“This can be applied in the design of culturally appropriate future research, and to support policy and practice towards stigma reduction, and improved mental care help-seeking in the Caribbean.”

**AUTHORS’ RESPONSE:** We thank the editor for this suggestion. We have incorporated this edit into the manuscript as shown in the Abstract-page 3 lines 46-48.

Methods:

According to the authors: “This systematic review addresses two research questions viz:

(1) What is the impact of mental health stigma on help-seeking in the Caribbean?

(2) What factors underlie the relationship between stigma and help-seeking in the Caribbean?

To achieve objectives (1) and (2), there is the need to undertake a process of triangulation of the data. I have read through the data extraction and appraisal, but I have not seen any mention of triangulation. Does it mean this was captured/presented in some other terms/terminologies?

I know that Triangulation facilitates validation of data through cross verification from more than two sources. Importantly, it also tests the consistency of findings obtained through different instruments and increases the chance to control, or at least assess some of the threats or multiple causes influencing our results. Triangulation will add great value to this paper.

**AUTHORS’ RESPONSE:** Thank you for raising this concern. To achieve objectives (1) and (2), a process of triangulation took place though a meta-synthesis of the quantitative and qualitative data, however we did not use the term triangulation in the original submission and express it in this way. To make it clearer for the reader, we have amended this sentence in the Method-Data synthesis section to make it explicit:

**“**Lastly, the data underwent a process of triangulation through a thematic meta-synthesis, where the findings from the quantitative and qualitative syntheses were integrated.” (page 9, lines 170-171).

Results - Studies with 3 participants (mentioned in the results section) should be excluded. It is not scientifically appropriate to use findings from 3 to 5 participants (even from qualitative)

**AUTHORS’ RESPONSE:** We thank the editor for their thoughtful response on this. As we are consolidating insights from an area where there is limited information from this geographical region, we believe it is important to include what literature is available to us. We acknowledge that studies with fewer participants may not be representative, however these studies have undergone a quality assessment, and we believe the findings should still be considered as such studies can provide rich, interesting data.

Additionally, the findings of this study aligned with the findings of the other qualitative studies included in this review where the data contributed to common themes generated from the thematic analysis (Making sense of mental health conditions’, ‘Anticipated/Experienced mental health experiences - illustrated in Supplementary Information - S4 Appendix Themes and subthemes with example participant quotations for included qualitative studies), and thus were not making a singular point.

We want to acknowledge in the manuscript that we recognise some of the included studies have small samples and the implications this may have, and have subsequently added the following extract into the Discussion-Limitations and strengths:

“Some of the included studies had small samples, thereby caution may be needed when interpreting the findings of this review. Nonetheless, the findings from studies with lower participant numbers did not drive any single conclusions drawn in this research but rather corroborated findings from other studies, and by including all eligible studies regardless of sample size this review was able to analyse and synthesise important and interesting data from the limited existing literature to provide a thorough evaluation.” (page 22 lines 406-411)

Results - I perceive that to arrive at an efficient conceptual model, it should be based on triangulation of the data from the meta-synthesis.

**AUTHORS’ RESPONSE:** Thank you for this comment. We agree that triangulation is required for an efficient conceptual model. The meta-synthesis is a result of triangulating the quantitative and qualitative data, however as we mention in a comment above, we previously did not explicitly state this. This how now been amended as demonstrated in the following sentence:

“The results from the quantitative and qualitative syntheses have been triangulated to produce an overall meta-synthesis.” (page 18, lines 313-314)

Discussion, future research - These sections are well written.

**AUTHORS’ RESPONSE:** We thank the editor for their positive feedback on these sections.

Implications for policy:

The authors said, “There is a need to incorporate mental health knowledge into early education and public awareness that includes stigma-reducing strategies”.

It seems not too logical connecting early education and public awareness to stigma reduction. I suggest that it would be more appropriate if we think of the interventions that involves community-based mental health education and health promotion that leverages public awareness on mental health, stigma and help-seeking.

**AUTHORS’ RESPONSE:** Thank you for this comment. We agree with the editor’s reflection and suggestion and have made the following edit:

‘There is a need for interventions that involve community-based mental health education and health promotion to contribute to public awareness on mental health, stigma and help-seeking." (page 21, lines 390-392)

**Reviewer’s Comments:**

[NOTE: If reviewer comments were submitted as an attachment file, they will be attached to this email and accessible via the submission site. Please log into your account, locate the manuscript record, and check for the action link "View Attachments". If this link does not appear, there are no attachment files.]

While revising your submission, please upload your figure files to the Preflight Analysis and Conversion Engine (PACE) digital diagnostic tool, <https://pacev2.apexcovantage.com/>. PACE helps ensure that figures meet PLOS requirements. To use PACE, you must first register as a user. Registration is free. Then, login and navigate to the UPLOAD tab, where you will find detailed instructions on how to use the tool. If you encounter any issues or have any questions when using PACE, please email PLOS at [figures@plos.org](mailto:figures@plos.org). Please note that Supporting Information files do not need this step.

**AUTHORS’ RESPONSE:** Thank you, we confirm this has been completed.
